# Possibilities and Limitations of the Sono-Fenton Process Using Mid-High-Frequency Ultrasound for the Degradation of Organic Pollutants

**DOI:** 10.3390/molecules28031113

**Published:** 2023-01-22

**Authors:** Efraím A. Serna-Galvis, Javier Silva-Agredo, Judy Lee, Adriana Echavarría-Isaza, Ricardo A. Torres-Palma

**Affiliations:** 1 Grupo de Investigación en Remediación Ambiental y Biocatálisis (GIRAB), Instituto de Química, Facultad de Ciencias Exactas y Naturales, Universidad de Antioquia UdeA, Medellín 050010, Colombia; 2 Grupo de Catalizadores y Adsorbentes (CATALAD), Instituto de Química, Facultad de Ciencias Exactas y Naturales, Universidad de Antioquia UdeA, Medellín 050010, Colombia; 3 School of Chemistry and Chemical Engineering, University of Surrey, Guildford GU2 7XH, UK

**Keywords:** contaminants degradation, Fenton reaction, opportunities and challenges, sonochemistry, water treatment

## Abstract

Mid-high-frequency ultrasound (200–1000 kHz) eliminates organic pollutants and also generates H_2_O_2_. To take advantage of H_2_O_2_, iron species can be added, generating a hybrid sono-Fenton process (sF). This paper presents the possibilities and limitations of sF. Heterogeneous (a natural mineral) and homogeneous (Fe^2+^ and Fe^3+^ ions) iron sources were considered. Acetaminophen, ciprofloxacin, and methyl orange were the target organic pollutants. Ultrasound alone induced the pollutants degradation, and the dual competing role of the natural mineral (0.02–0.20 g L^−1^) meant that it had no significant effects on the elimination of pollutants. In contrast, both Fe^2+^ and Fe^3+^ ions enhanced the pollutants’ degradation, and the elimination using Fe^2+^ was better because of its higher reactivity toward H_2_O_2_. However, the enhancement decreased at high Fe^2+^ concentrations (e.g., 5 mg L^−1^) because of scavenger effects. The Fe^2+^ addition significantly accelerated the elimination of acetaminophen and methyl orange. For ciprofloxacin, at short treatment times, the degradation was enhanced, but the pollutant complexation with Fe^3+^ that came from the Fenton reaction caused degradation to stop. Additionally, sF did not decrease the antimicrobial activity associated with ciprofloxacin, whereas ultrasound alone did. Therefore, the chemical structure of the pollutant plays a crucial role in the feasibility of the sF process.

## 1. Introduction

Hybrid systems, where two or more advanced oxidation processes (AOPs) are applied simultaneously, are known to promote the effective degradation of pollutants [1]. The sono-Fenton process (sF) is a very popular hybrid AOP that combines the Fenton reaction with ultrasound waves [US, 20–2000 kHz, represented by the symbol “)))”] to deal with recalcitrant contaminants. The ultrasound-based process involves using the acoustic cavitation phenomenon, i.e., the formation, growth, and implosive collapse of microbubbles in aqueous media, to produce chemical effects. The implosion of micro-bubbles generates localized hot spots with transient temperatures up to 5000 K and pressures ~1000 atm. Such extreme conditions lead to the cleavage of water molecules and dissolve oxygen into radicals (Equations (1)–(4)). These radical species can either react with pollutants within the bubble or in the bubble-liquid interface. Large nonvolatile and very hydrophilic molecules cannot enter the cavitation bubbles or come close to the interfacial zone, and consequently, they react slowly with a few radicals that reach the solution bulk [2]. In addition to the reaction with the pollutants, some sonogenerated hydroxyl radicals can recombine, leading to the accumulation of H_2_O_2_ (Equation (5)) [3]. In turn, the Fenton process involves reactions of peroxides (usually hydrogen peroxide) with iron ions to form active oxygen species (such as HO•) that oxidize organic pollutants (Equations (6) and (7)) [4].

H_2_O + ))) → •H + HO•, (1)

O_2_ + ))) → 2 •O, (2)

H_2_O + •O → 2 HO•, (3)

O_2_ + •H → •O + HO•, (4)

2HO• → H_2_O_2_, (5)

Fe^2+^ + H_2_O_2_ → Fe^3+^ + HO• + HO^−^, (6)

Fe^3+^ + H_2_O_2_ → Fe^2+^ + HOO• + H^+^, (7)

The hybrid sF process takes advantage of H_2_O_2_ accumulated from the HO• recombination during the ultrasonic treatment (Equation (5)) to promote Fenton-type reactions (Equations (6) and (7)) enhancing the degradation kinetics, minimizing the use of reagents (iron and hydrogen peroxide), and thus limiting secondary pollution and costs [5].

Diverse experimental configurations are possible for the sF process. Low (20–150 kHz) and mid-high (200–2000 kHz) frequency ultrasound reactors can be used [2,6,7,8]. It should be mentioned that at low-ultrasound frequencies there is a low generation of hydroxyl radicals, and consequently, a small accumulation of H_2_O_2_ (particularly very low at low applied power, i.e., <100 W). Then, hydrogen peroxide from an external source should be added to the reaction systems [6,7].

The sF systems can also use homogeneous or heterogeneous iron sources. For homogeneous systems, salts such as FeCl_3_× 6H_2_O and FeSO_4_× 7H_2_O are typically utilized [9]. Meanwhile, for the heterogeneous systems, Fe_3_O_4_ magnetic nanoparticles [9], Fe_3_O_4_/ZnO/graphene nanocomposites [10], pyrite nanorods [11], Fe_2_O_3_ on SBA-15 mesoporous silica [8], zero-valent iron (ZVI) [12,13], and iron-containing zeolites [2] or iron oxides supported on zeolites [14], among others, have been evaluated. It is reported that the ultrasonic component can decrease the mass transfer limitations for solid–liquid heterogeneous systems [9].

Many studies report successful application of the sF process to degrade organic pollutants. However, most of this research primarily focuses on the hybrid sF process using low ultrasound frequency (i.e., below 150 kHz) [6,9,10,11,12,13,14,15,16,17]. Furthermore, since most studies consider only one pollutant, the role of the pollutant’s nature in the sF process is not well examined [2,8,18]. Therefore, we developed systematic research, applying mid-high ultrasound frequency (200–1000 kHz) to degrade three representative pollutants, i.e., an antibiotic (ciprofloxacin), an analgesic (acetaminophen), and a dye (methyl orange), to evaluate the role of the pollutant’s nature. Both heterogeneous and homogeneous sF systems were considered. For the heterogeneous system, a natural mineral from Colombia was tested. This material was selected because of its high feasibility for use in Fenton-based systems according to previous research [19]). Ferrous and ferric salts were employed for the homogeneous sources. Special attention was paid to the interaction of the pollutant with iron, the decrease of biological activity, and the primary transformations experienced by the pollutants. Moreover, the advantages and limitations of the different sF configurations are discussed.

## 2. Results and Discussion

### 2.1. Suitable Conditions for the Operation of the Ultrasound Reactor to Produce H_2_O_2_

**Figure 1:**
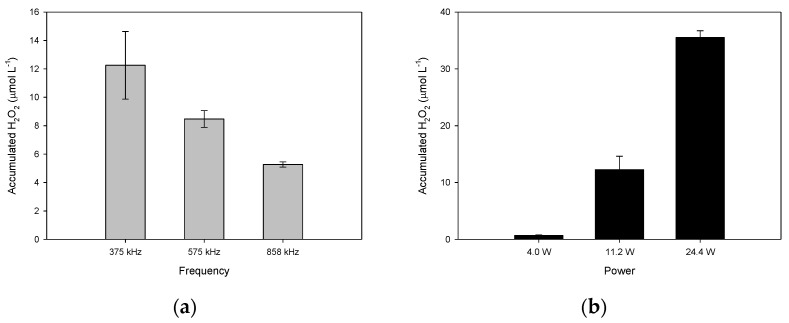
Capability of the ultrasound reactor to produce H_2_O_2_ in distilled water at 20 min of sonication. (**a**) Effect of the ultrasound frequency (V: 250 mL, P: 11.2 W); (**b**) Effect of ultrasound power (V: 250 mL, f: 375 kHz).

Initially, the capability of the sonochemical reactor to produce hydrogen peroxide (Equation (5)) in distilled water at different frequencies (375, 575, 858 kHz) was established. From Figure 1a, it can be noted that, as the ultrasound frequency increased, the accumulation of H_2_O_2_ diminished. In general, the size of the bubble decreases as the ultrasonic frequency increases [20,21]. Hence, at high frequencies (e.g., 858 kHz), the cavitation bubbles collapse so quickly that they do not achieve maximum size; this decreases the production of hydroxyl radicals [22], and a low level of hydrogen peroxide formation is observed. Therefore, the accumulation of H_2_O_2_ is more favored at 375 kHz than at 858 kHz.

At 375 kHz of frequency, the effect of the actual acoustic power on the accumulation of H_2_O_2_ was evaluated. As seen in Figure 1b, more hydrogen peroxide accumulated as the power was augmented from 4.0 to 24.4 W. In the literature, it is proposed that, at high power, the bubble could expand more during the rarefaction stage of the acoustic cycle and increase the bubble radius, allowing the bubble to cavitate, which can also increase the population of cavitation bubbles [20,21]. Furthermore, as the acoustic power is augmented, more violent cavitation events occur [23]. Consequently, more radicals are formed at higher acoustic power values, leading to a higher H_2_O_2_ accumulation. Considering the results in Figure 1, 375 Hz and 24.4 W were selected as suitable operational conditions to perform the sF process.

### 2.2. Heterogeneous Sono-Fenton Processes for the Elimination of Pollutants

**Figure 2:**
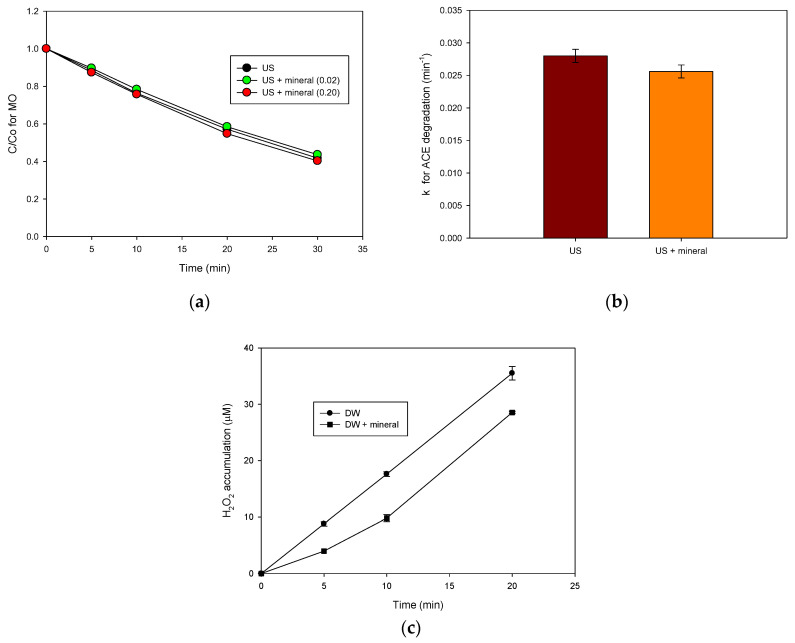
Heterogeneous sono-Fenton for degrading organic pollutants. (**a**) Effect of mineral concentration on the MO degradation; (**b**) Effect of the natural mineral (at 0.20 g L^−1^) on ACE degradation; (**c**) Hydrogen peroxide evolution in distilled water in the absence (DW) and presence of the solid iron source (DW + mineral) at 0.20 g L^−1^. Experimental conditions: f: 375 kHz, P: 34.4 W, [MO]_initial_ = [ACE]_initial_: 30.6 µmol L^−1^, pH_initial_: 5.6, and V: 250 mL.

After determining the suitable operational conditions for the hydrogen peroxide sonoproduction, the conditions were applied through ultrasound alone and with the sF process, using the natural mineral as a heterogenous source of iron at two concentrations (0.02 and 0.20 g L^−1^, Figure 2a) and starting with MO as a model organic pollutant. The sonochemical process alone degraded ~56% of the pollutant at 30 min of treatment. However, the addition of the natural mineral did not enhance the MO degradation. At the two concentrations of the natural solid, the pollutant evolution was very close to that obtained in its absence. It is important to mention that MO is not adsorbed on the mineral (Figure S1 in the Supplementary Materials), which is explained by the very low surface area (19.79 m^2^ g^−1^) of this solid material. Furthermore, if the MO pollutant is replaced by ACE (Figure 2b), a low effect of the solid on the pseudo-first-order kinetic constants for the treatments was observed. Similar to the results observed for MO, ACE was not adsorbed on the mineral surface (Figure S2a).

We should mention that another work in the literature reports that the addition of a heterogenous iron source to high-frequency ultrasound improves the degradation of organic pollutants (Table 1). However, such a system only truly works if a high concentration of H_2_O_2_ from an external source is also added at the beginning of the process [8]. MO and ACE are non-volatile and soluble compounds; thus, they are degraded by the sonogenerated hydroxyl radicals that reach the solution bulk. Concomitant to the interaction of these pollutants with HO•, the recombination of radicals leads to the formation of hydrogen peroxide (Equation (5)). The interaction of the heterogeneous iron source with hydrogen peroxide is therefore expected [24]. Moreover, the solid particles could promote the degradation of pollutants by providing additional nuclei for the formation of cavitation bubbles. However, the attenuation of the ultrasound waves by the particles may have adverse effects, which could reduce the degradation of the pollutant. Then, the net effect is dependent on the ultrasound system and solid material [2,25,26,27,28].

To better understand the role of the tested solids, a control experiment (distilled water without pollutants) was carried out. The evolutions of the sonogenerated H_2_O_2_ in the presence and absence of the natural mineral were compared (Figure 2c). It was found that the hydrogen peroxide accumulation was lower when the solid was present than in its absence. The decrease of the H_2_O_2_ concentration can therefore be associated with the quenching of waves by the presence of the solid particles of the mineral and/or the interaction among H_2_O_2_ and the ferric (=Fe^3+^) or ferrous species (=Fe^2+^) in the mineral (Figure S2b presents the XRD pattern of this mineral, demonstrating the predominance of hematite “Fe_2_O_3_”, with some traces of siderite “FeCO_3_”), which can produce degrading radicals (Equations (8) and (9)).

=Fe^2+^ + H_2_O_2_ → =Fe^3+^ + HO• + HO^−^, (8)

=Fe^3+^ + H_2_O_2_ → =Fe^2+^ + HOO• + H^+^, (9)

It is also important to consider that small amounts of iron could be leached from the solid. Indeed, in our research team’s previous work regarding the use of this natural mineral in a photo-Fenton system, we found that less than 0.1 mg L^−1^ is leached from the solid material [19]. Thereby, in the sono-Fenton system, the involvement of the homogenous component of Fenton (Equations (6) and (7)) is plausible, and this also contributes to the decrease in the H_2_O_2_ concentration observed in Figure 2c.

Hence, the results of degradation in Figure 2 suggested that, despite the solids which may induce some attenuation of the ultrasound waves, the reaction system in the presence of the iron species in the solid particles and the leached iron can generate enough radicals to degrade the target pollutant. A balance among the contrary phenomena is proposed to explain the similar pollutants degradations in the absence and presence of a heterogeneous iron source (i.e., the Colombian natural mineral). Furthermore, these results also indicate that the sonochemical processes could be applied to treat polluted water even if it contains a high concentration of suspended solids (e.g., 0.20 L^−1^).

However, when this work is compared with other reports in the literature (Table 1), it can be noted that our results were similar to those reported for the heterogeneous sono-Fenton process at mid-high-frequency (e.g., 850 kHz) [2]. Moreover, results in the literature also show that the performance of the sono-Fenton at mid-high-frequency can be enhanced by the addition of H_2_O_2_. At low frequencies (<150 kHz), the physical effects of ultrasound (which are stronger than at high frequencies) play a relevant role, favoring the iron leaching, increasing the turbulence and mass transfer, and promoting particle disaggregation which augments the active sites on the catalyst surface (Table 1).

### 2.3. Homogeneous Sono-Fenton to Degrade Diverse Organic Pollutants

#### 2.3.1. Effect of Iron (II) Concentration and Iron Species (II or III)

**Figure 3:**
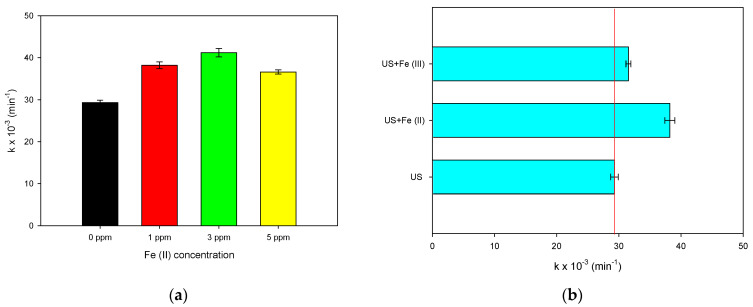
Homogeneous sono-Fenton for degrading MO as the probe compound. (**a**) Effect of iron (II) ions concentration; (**b**) Effect of the iron species (II or III, at 1 ppm (mg L^−1^)). Experimental conditions: f: 375 kHz, P: 34.4 W, [MO]_initial_: 30.6 µmol L^−1^, pH_initial_: 5.6, and V: 250 mL.

The other strategy to perform an sF process is the addition of soluble iron salts (e.g., iron sulfates or iron chlorides) obtaining a homogeneous system. Herein, the homogeneous sF involving ferrous ion (from FeSO_4_× 7H_2_O) was assessed first. Three different amounts of Fe^2+^ (1.0, 3.0, and 5.0 mg L^−1^) were added to the sonochemical reactor, and the sF process was developed using MO as the probe molecule (Figure 3a). The presence of ferrous ions at 1 mg L^−1^ augmented the pseudo-first-order rate constant (k) regarding the system with no iron, and when the Fe^2+^ was increased up to 3 mg L^−1^, a higher acceleration of the pollutant degradation was observed. However, if the ferrous iron concentration is 5 mg L^−1^, the k-value is lower than the one obtained at 1 mg L^−1^.

The presence of ferrous ions in the solution bulk of the sonochemical system promotes the production of extra hydroxyl radicals through the Fenton reactions with the sonogenerated H_2_O_2_ (Equations (5) and (6)), improving the degradation of the pollutants [2] according to Figure 3a. As the Fe^2+^ concentration increases, higher production of radicals and degrading effects are seen. Nonetheless, an excess of ferrous ions (e.g., 5 mg L^−1^) induced a scavenging interaction between iron and hydroxyl radical (Equation (10), [4,29]), and the radicals are consumed, competing with the pollutant degradation. Thereby, the sF process at low or moderate concentrations of ferrous ions has excellent possibilities, but at a high Fe^2+^ concentration, its ability to degrade pollutants could be limited.

Fe^2+^ + HO• → Fe^3+^ + HO^−^, (10)

Once the effect of the iron (II) concentration on the sF process was established, the treatment of MO as a probe pollutant, under the substitution of ferrous ions by ferric ions, was performed. Figure 3b compares the pseudo-first-order kinetics constants (k-values) for the treatment of MO by sF using Fe^2+^ or Fe^3+^. The ferric ion also improved the degradation kinetics of the pollutant, but the enhancing effect induced by Fe^2+^ is superior to that obtained when Fe^3+^ is added (Figure 3b). Such findings are explained by considering the interaction between the two iron species with hydrogen peroxide and the formed radicals, respectively. The reaction of Fe^2+^ with H_2_O_2_ (Equation (6), k: 53–76 M^−1^ s^−1^) is faster than the Fe^3+^-H_2_O_2_ interaction (Equation (7), k < 10^−2^ M^−1^ s^−1^) [29,30,31]. The former reaction (Equation (6)) also produces the hydroxyl radical (E°: 2.73 V, [4]), and this oxidizing agent is stronger than hydroperoxyl radical (E°: 1.44–1.65 V, [32]), which is formed from the Fe^3+^-H_2_O_2_ interaction (Equation (7)).

#### 2.3.2. Degradation of Diverse Organic Pollutants by Homogeneous Sono-Fenton

**Figure 4:**
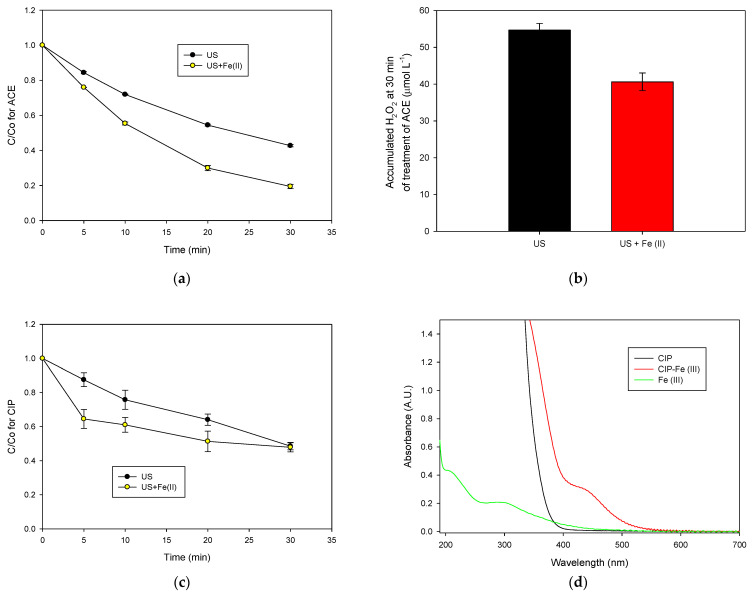
Treatment of ACE and CIP by the homogeneous sono-Fenton process (**a**) Degradation of ACE; (**b**) H_2_O_2_ accumulation during the treatment of ACE; (**c**) Degradation of CIP, (**d**) Experimental evidence based on the UV-Vis spectrum for the formation of the CIP-Fe^3+^ complex. Experimental conditions: f: 375 kHz, P: 34.4 W, [ACE]_initial_: [CIP]_initial_: 30.6 µmol L^−1^, [Fe^2+^]: 1.0 mg L^−1^, pH _initial_: 5.6, and V: 250 mL.

The results in the previous sections showed that soluble salts of iron (II) at low concentrations are more convenient to obtain positive effects on the sF process. Therefore, such conditions (1.0 mg L^−1^ of Fe^2+^) were used to treat other organic pollutants aiming to evaluate the effect of the nature of the contaminant on the performance of the process. The degradation of the pharmaceuticals ACE and CIP were considered (Figure 4). Figure 4a compares the ACE evolution under the action of ultrasound alone and with sF. The addition of soluble iron (II) to the sonochemical reaction increased the ACE degradation from 43% to 80% (after 30 min of treatment) compared to the action of ultrasound alone. To better support the role of the ferrous ions in the system, the accumulation of H_2_O_2_, after 30 min of ACE treatment in the absence and presence of the ferrous ions was measured.

As seen in Figure 4b, the accumulation of hydrogen peroxide in the sF process (US + Fe (II)) is lower than in the sonochemical system alone (US), indicating the generation of extra radicals by the Fenton reaction (Equations (6) and (7)), which is responsible for the acceleration of the ACE degradation. In turn, Figure 4c depicts the case of CIP treatment using the sonochemical system and sF process. In contrast to the results observed for ACE or MO, the addition of iron (US + Fe (II), sF) improved the antibiotic degradation in the first 5 min of treatment, but the pollutant elimination stopped after this. Even at 30 min after the process began, the removals of CIP by sonochemistry and sF were the same. In the sF system, it is possible that the Fe^3+^, which is produced from the Fenton reaction (Equation (6)), interacted with the non-degraded molecules of CIP, thus limiting the iron availability.

As shown in Figure 4d, the interaction of ferric ions with CIP produced a new band between 400 and 600 nm in the UV-Vis spectrum. Moreover, the mixture of the antibiotic and the ferric ions induced an intense yellow coloration of the solution. These results evidenced the formation of a CIP-Fe^3+^ complex [33]. However, no interaction between ferrous ions and CIP was observed (Figure S3). Recent studies have also reported that the interaction between fluoroquinolones (CIP belongs to this antibiotics class) and ferric ions leads to the formation of stable complexes [33,34]. It can be noted that CIP has the keto-carbonyl moiety (structure with a higher number of lone electron pairs), which favors the interaction between this fluoroquinolone and ferric ion through chelation [35,36,37]. This contrasts with the case of ACE, which is not able to form complex iron ions. In fact, the presence of ferric or ferrous ions in the ACE solution did not generate new adsorption bands, as supported by the UV-Vis spectra in Figure S4.

Therefore, the results in Figure 4b could be explained by considering that, at the beginning of the CIP treatment by sF, the ferrous ions reacted with the sonogenerated H_2_O_2_ inducing an acceleration of the pharmaceutical degradation and producing ferric ions (Equation (6)). Subsequently, the ferric ions are complexed by the remaining molecules of CIP. The formed complex is a charged hydrophilic molecule [33,38] that is placed far away from the cavitation bubble and the hydroxyl radicals. Consequently, the complex is recalcitrant to the sonochemical action, and the CIP concentration remains constant, as observed in Figure 3b. Thus, the above results from the degradation of CIP, ACE, and MO by the sF system indicate that the nature of the pollutant strongly influenced the process performance. Thereby, sF is more suitable for degrading organic contaminants that have no complexation capability toward the iron species.

A comparison of the homogeneous sono-Fenton process with the existing literature (Table 2) shows that our system had similar results to those obtained using mid-high-frequency ultrasound by the single addition of Fe^2+^, which accelerates the elimination of the contaminant. It is also reported that an excess of ferrous ions induces scavenging effects. From information available in the literature, it can also be noted that the performance of the sono-Fenton process at high frequencies is improved by the external addition of H_2_O_2_. Other studies indicate that low-frequency ultrasound in combination with Fe^2+^ and H_2_O_2_ is also useful for degrading organic contaminants, even presenting better results than Fenton systems or ultrasound alone. Most studies utilize ferrous ions, and a few of them report that ferric ions have a lower enhancing rate in eliminating pollutants. Additionally, the complexation of the target pollutant with iron species is not reported or discussed in the previous literature about the sono-Fenton process (Table 2).

### 2.4. A Strategy for the Treatment of CIP

As shown in Section 2.3.2, the sF system was not able to degrade CIP because of the production of an organo-complex stable to the sonochemical action. Therefore, considering the capability of the formed complex to absorb ultraviolet light (Figure 4d), the photo-treatment was tested using very energetic irradiation (i.e., UVC light at 254 nm). This type of light was selected for its ability to photolyze H_2_O_2_ (Equation (11)) and promote the photo-reduction of aqua-complex of ferric ions (Equation (12)), producing hydroxyl radicals profitable for the degradation of the pollutant [41,42]. Hence, after 20 min of application of the sF process, the resultant solution was removed from the ultrasound reactor, transferred into a beaker, and subsequently irradiated using UVC light (a sequential treatment, i.e., sF followed by the UVC action).

H_2_O_2_ + *hv_254 nm_* → 2 HO•, (11)

Fe^3+^ + H_2_O + *hv_254 nm_* → Fe^2+^ + HO• + H^+^ (12)

**Figure 5:**
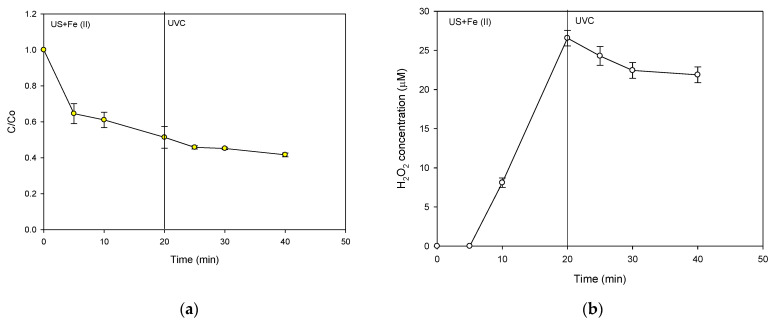
Sequential application of sF and UVC. (**a**) Evolution of CIP under the action of US followed by UVC irradiation; (**b**) Evolution of the H_2_O_2_ accumulation during the CIP treatment under the action of US followed by UVC irradiation. Experimental conditions: f: 375 kHz, P: 34.4 W, [CIP]_initial_: 30.6 µmol L^−1^, [Fe^2+^]: 1.0 mg L^−1^ (18.0 µmol L^−1^), pH_initial_: 5.6, V: 250 mL, and UVC: 3.02 × 10^17^ photons s^−1^.

From Figure 5a, it can be noted that the treatment using ultraviolet irradiation had a low degrading action on the complexed CIP. the H_2_O_2_ evolution presented in Figure 5b also indicated a low consumption of hydrogen peroxide. The low photodegradation of the complexed CIP can be associated with the relocation of part of the electron density from the organic structure (i.e., CIP) on the metal ion [33,43]. Indeed, previous theoretical works have reported that the interaction of fluoroquinolones with the metal cations increases the activation energy for some photo-transformation pathways, thus making the complex more recalcitrant than the free antibiotic to the light action [43,44,45]. Furthermore, as the complex had strong adsorption of the UVC light, this may affect the production of radicals by the hydrogen peroxide photolysis (Equation (11)) or the photo-reduction of aqua-complex of ferric ions (Equation (12)). Hence, the degradation of the complexed CIP and the H_2_O_2_ consumption are low under the UVC light action, as observed in Figure 5.

**Figure 6:**
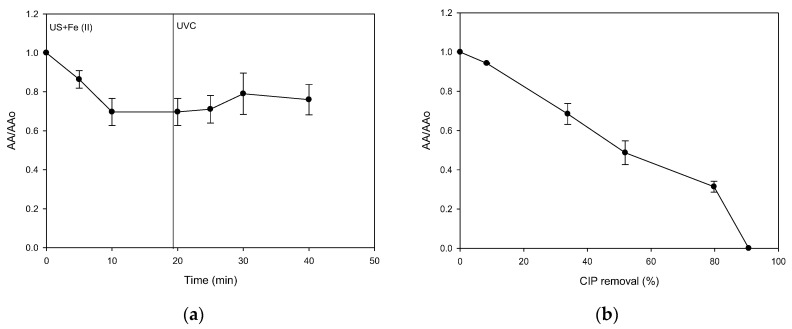
Evolution of the antimicrobial activity (AA). (**a**) Evolution of AA associated with CIP under the action of sF (US+ Fe (II)) followed by UVC irradiation; (**b**) Evolution of AA associated with CIP under the action of the sonochemical process alone. Experimental conditions: f: 375 kHz, P: 34.4 W, [CIP]_initial_: 30.6 µmol L^−1^, [Fe^2+^]: 1.0 mg L^−1^ (18.0 µmol L^−1^), pH_initial_: 5.6, V: 250 mL, and UVC: 3.02 × 10^17^ photons s^−1^.

To analyze the treatment extent, the antimicrobial activity (AA) evolution corresponding to the sequential treatment (sF followed by UVC) was also stated (Figure 6a). It can be noted that the initial ultrasound step led to a decrease in the AA (~30% after 20 min of treatment). However, the photochemical component had no significant effect on the AA decrease. These results regarding the AA evolution were consistent with those observed in Figure 5. As the elimination of the antibiotic stopped, the AA remained approximately constant. Hence, the decrease in antimicrobial activity was related to the diminution in the concentration of CIP. Moreover, it should be remarked that the sequential system was unable to decrease the AA completely.

**Figure 7:**
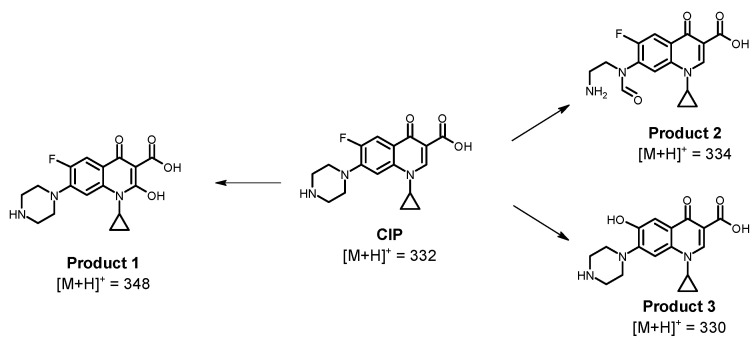
Primary transformation products for CIP under the action of ultrasound process alone. The mass spectra of CIP and its primary transformation products are presented in Figures S5–S8.

As the sF process is limited, the capability of the ultrasound system alone to decrease the AA associated with CIP was also assessed (Figure 6b). This process achieved a complete decrease in AA. Indeed, at 90% of CIP removal, the AA was 100% decreased, suggesting that the residual CIP amount (i.e., ~3.1 µmol L^−1^) is below its minimum inhibitory concentration (MIC), and the degradation products could have lower AA than the parent antibiotic because of structural modifications on the antibiotic, which would be induced by the action of the process [38]. Therefore, to better understand the AA evolution under the US action, the primary transformation products were elucidated (Figure 7).

The sonochemical action induced a hydroxylation of the quinolone nucleus (Product 1), cleavage plus oxidation of the piperazyl ring (Product 2), and substitution of the fluorine on CIP (Product 3, Figure 7). These products come from the attacks of the sonogenerated hydroxyl radicals. They have also been detected during the treatment of CIP by other oxidative processes, such as pulsed radiolysis [46], photocatalysis using bismuth oxybromide [47], electrochemical [48], and persulfates-based systems [49,50], in addition to photolytic and photocatalytic treatments [51]. The modifications on the quinolone (Product 1) and piperazyl (Product 2) rings may alter the acid/base speciation and decrease the lipophilicity and the cell permeability [51], thus diminishing the AA. In turn, the fluorine replacement on the CIP structure (as shown for Product 3) could also diminish the antimicrobial action as the fluorine atom on CIP plays a determinant role in the cell permeation [51] as well as inhibiting the DNA gyrase (which is the action mode of this antibiotic on bacteria [52]). Such structural transformations on CIP by the sonochemical explain the AA decrease observed in Figure 6b. Therefore, the sF has limited performance for eliminating ciprofloxacin, and it is more convenient for this antibiotic treatment to use ultrasound alone.

## 3. Materials and Methods

### 3.1. Reagents

Ciprofloxacin (CIP) and acetaminophen (ACE) were provided by Laproff laboratories (Medellín, Colombia). Acetonitrile, hydrochloric acid, Iron (III) chloride hexahydrate, Iron (II) sulfate heptahydrate, methyl orange (MO), and nutrient agar were purchased from Merk (Darmstadt, Germany). Formic acid was obtained from Carlo Erba (Barcelona, Spain). Peptone and yeast extract powders were purchased from Oxoid (Basingstoke, UK). All the culture media and broths were sterilized at 121 °C using an autoclave. All solutions were prepared using distilled water.

As the heterogeneous iron source, a natural mineral was used. This material was obtained from an iron mine in Colombia (Duitama, Boyacá). It was used without any pretreatment. The specific surface area was estimated at 19.79 m^2^ g^−1^ by the Brunauer–Emmett–Teller (BET) theory, and N_2_ physisorption measurements on a Micromeritics 3Flex apparatus were used for the measurements. The natural mineral contained 81.3% by mass of iron (as iron oxides, mainly hematite with traces of siderite, see Figure S2b) [19].

### 3.2. Reaction System

**Figure 8:**
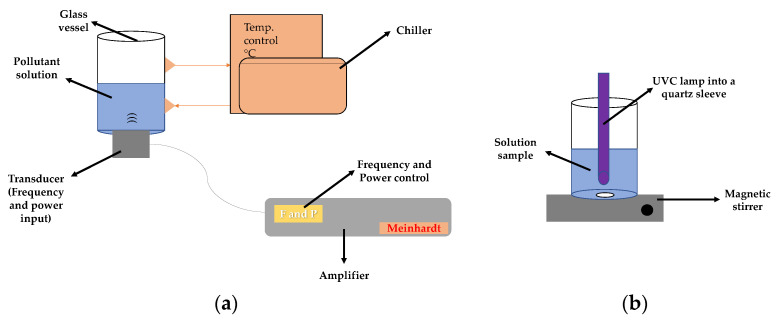
Scheme of the experimental setups. (**a**) Ultrasound reactor for the sono-Fenton process, in this reactor, frequency and power can be adjusted. (**b**) Reactor for the photo-treatment.

Sonochemical experiments were carried out in a high-frequency Meinhardt Ultrasonics reactor (i.e., >200 kHz) equipped with a cylindrical glass vessel containing 250 mL of pharmaceutical solution. The ultrasound waves were emitted from a transducer (with the possibility of operation at three different frequencies: 375, 575, and 858 kHz) placed at the bottom of the reactor. In this reactor, both frequency and power can be changed (Figure 8a). The actual ultrasound power was determined by the calorimetric method [53].

For the photo-treatment of the solutions previously treated by ultrasound, a homemade reflective reactor was used that was equipped with three Osram Puritec (HNS G5, 60 W) UVC lamps (Wilmington, MA, USA) and a main emission peak at 254 nm. A beaker containing 250 mL of the sample that was under constant magnetic stirring was submitted to the UVC action (Figure 8b). The actual photon flux of UVC light in the reactor was 3.02 × 10^17^ photons s^−1^ (determined by actinometry using ferrioxalate [54]).

### 3.3. Analyses

The evolution of the pharmaceuticals during treatments was followed by using a UHPLC Thermo Scientific (Waltham, MA, USA) Dionex UltiMate 3000 instrument equipped with an Acclaim™ 120 RP C18 column (5 µm, 4.6 mm × 150 mm) and a diode array detector. The chromatographic conditions of each pharmaceutical, such as composition mobile phase, flow, and detection wavelength, are detailed in Table 3. In the case of the methyl orange, its degradation was followed by measuring the absorbance at 465 nm using a UV5 Mettler-Toledo spectrophotometer. During the pollutants’ treatment, samples of 0.5 mL were periodically taken from the reaction systems (the total taken volume was always lower than 10% of the initial volume in each system). The degradations fitted well to pseudo-first-order kinetics, and the rate constants (k) were obtained as the slope of the ln (C/Co) vs. time plots, as illustrated in Figure S9. Table S1 summarizes the k-values associated with the degradation of the target pollutants, with their corresponding errors and correlation coefficients.

Accumulation of sonogenerated hydrogen peroxide was estimated by iodometry [40]. An aliquot of 600 µL from the reactors was added to a quartz cell containing 1350 µL of potassium iodide (0.1 M) and 50 µL of ammonium heptamolybdate (0.01M). After 5 min, the absorbance at 350 nm was measured using a spectrophotometer (UV5 Mettler Toledo).

The evolution of the antimicrobial activity of CIP was analyzed by the diffusion agar method [55], using 30 µL of the sample, *S. aureus* (ATCC 25923) as the indicator microorganism, and the incubation was performed at a Memmert incubator at 37 °C during 24 h.

The primary transformation products were established using an HPLC Agilent 1200 series coupled to an Agilent LC/MSD VL SQ mass spectrometer (Santa Clara, CA, USA). The column and mobile phase were operated under the same conditions presented in Table 3. The injection volume was 10 µL and the mass spectrometer detector was operated in positive ion mode [56].

XRD analysis for the natural mineral was carried out in an X’Pert MPD PRO from PANalytical (Malvern, UK) apparatus using Cu Kα radiation at a grazing incident angle of 4°. The sample was sieved to separate the large aggregates and avoid X-ray reflection due to size. It was then suspended in MQ-water and dropped/fixed on a glass slide [19].

## 4. Conclusions

The heterogeneous iron source had a low effect on the degrading action of the sonochemical process because, despite the solid particles of the natural mineral could induce some attenuation of the ultrasound waves, the system can generate enough radicals to degrade the target pollutants. In the homogenous sF performance, a strong dependence on both the oxidation state and concentration of iron was observed, where the use of ferrous ions, at relatively low concentrations, promotes the formation of extra hydroxyl radicals beneficial to enhance the degradation of the pollutants. Finally, it must be considered that the interaction of the pollutants with the iron species can alter the degrading action. In fact, the formation of ferric complexes makes some pollutants recalcitrant to the action of ultrasound or UVC light. In this last case, it is convenient to utilize ultrasound alone, which can efficiently degrade the non-complexed pollutant. In fact, in the case of antibiotics such as CIP, the ultrasound alone is even able to decrease the antimicrobial activity thanks to transformations induced by the sonochemical process to the parent pollutant.

## Figures and Tables

**Table 1:** Comparison of the tested heterogeneous sono-Fenton system with other cases reported in the literature.

Heterogeneous Iron Source	Ultrasound System	Target Pollutant	Main Results	Reference
Fe_2_O_3_/SBA-15 (0.6 g L^−1^)	20, 382, 584, and 1142 kHz, with external addition of H_2_O_2_ (1.19 g L^−1^*)*	Phenol	Highest elimination of aromatic compounds and mineralization at 584 kHz due to its highest acoustic power and elevated production of degrading radicals. Iron ions are leached from the solid catalyst.	[8]
ZSM-5 zeolite containing iron (0.1 mmol L^−1^ of iron)	850 kHz, with the external addition of H_2_O_2_ (5.0 mmol L^−1^)	Orange II	The degradation of Orange II induced by the sono-Fenton system was very similar to that obtained under the combination of ultrasound with hydrogen peroxide.	[2]
4A-zeolite supported α-Fe_2_O_3_ (0.5 g L^−1^)	40 kHz	Orange II	The removal of the pollutant is related to adsorption on the catalyst, heterogeneous, and homogeneous (iron dissolved into the solution) Fenton reaction.	[14]
Zero valent iron-ZVI (0.5 g L^−1^)	28 kHz, without and with external addition of H_2_O_2_ (30–100 µmol L^−1^)	Orange G	Ultrasound promotes the leaching of iron ions, which enhances the dye degradation regarding ultrasound alone or adsorption on the ZVI. Moreover, the external addition of H_2_O_2_ increases the degradation and mineralization. However, an excess of H_2_O_2_ induces scavenging effects.	[13]
Zero valent iron-ZVI (1.0 g L^−1^)	60 kHz, with the external addition of H_2_O_2_ (10.3 mmol L^−1^)	Reactive Black 5	Synergistic effects for the dye degradation by the ZVI/H_2_O_2_/ultrasound combination. Hydrogen peroxide produced from sonolysis in contact with Fe (II), coming from ZVI corrosion, triggers the Fenton reaction.	[12]
Pyrite nanorods (0.6 g L^−1^)	40 kHz, with the external addition of H_2_O_2_ (1.0 mmol L^−1^)	Reactive Blue 69	The sono-elimination of the target dye is significantly improved by the addition of pyrite nanorods and H_2_O_2,_ reporting synergy for the combination of ultrasound with pyrite and hydrogen peroxide. Synergy is explained considering that ultrasound waves increase the turbulence and mass transfer and also promote particle disaggregation by augmenting the active sites on the catalyst surface. In turn, the crevices of the solid particles act as cavitation nuclei.	[11]
Fe_3_O_4_/ZnO/graphene composites	40 kHz is added to a Fenton process	Methylene blue and Congo-red	The addition of ultrasound irradiation to the Fenton process improves the degradation of both dyes.	[10]
Fe_3_O_4_ magnetic nanoparticles (0.585 g L^−1^)	40 kHz, with the external addition of H_2_O_2_ (160 mmol L^−1^)	Bisphenol-A	No adsorption of the pollutant on the catalyst. The decomposition of H_2_O_2_ into radicals promoted by ultrasound plus disaggregation of particles favors the Fenton reaction, leading to synergistic effects on the degradation of bisphenol-A.	[9]
Natural mineral containing iron oxides, mainly hematite (Fe_2_O_3_) (0.20 g L^−1^)	375 kHz	MO and ACE	Degradation of the pollutants by sono-Fenton was very close to that obtained by ultrasound alone	This work

**Table 2:** Comparison of the tested homogeneous sono-Fenton system with other cases reported in the literature.

Homogeneous Iron Source	Ultrasound System	Target Pollutant	Main Results	Reference
Fe^2+^ (10 mg L^−1^)	35 and 53 kHz, with the external addition of H_2_O_2_ (50 mg L^−1^)	Reactive Blue 181	The sono-Fenton process has superior performance compared to the Fenton system in terms of degrading the target pollutant because of the production of some oxidizing agents as a result of sonication.	[6]
Fe^2+^ (3.0 mg L^−1^)	20 kHz, with the external addition of H_2_O_2_ (0.5 mmol L^−1^)	Reactive Blue 19	The combination of ultrasound with Fe^2+^ and H_2_O_2_ leads to a higher degradation of the dye than the individual components (even more than the Fenton system) of the sono-Fenton process.	[16]
Fe^2+^ (0.134 mmol L^−1^)	20 kHz, with the external addition of H_2_O_2_ (6.4 mmol L^−1^)	Ibuprofen	The addition of Fe^2+^ and H_2_O_2_ to the ultrasound reactor increases both the degradation and mineralization of the pharmaceutical.	[18]
Fe^2+^ (Different concentrations)	850 kHz, without and with the external addition of H_2_O_2_ (Diverse concentrations)	Orange II	Acceleration of the pollutant degradation by adding Fe^2+^, taking advantage of the sono-generated H_2_O_2_. The external addition of both Fe^2+^ and H_2_O_2_ lead to the best dye degradation. However, an excess of Fe^2+^ and H_2_O_2_ leads to scavenging effects.	[2]
Fe^2+^ (0.1 mmol L^−1^)	300 kHz	Bisphenol-A	The degradation and mineralization of bisphenol-A are enhanced by the presence of ferrous ions due to the Fenton reaction using the H_2_O_2_ coming from the sonochemical system.	[39]
Fe^2+^ (90 µmol L^−1^)	600 kHz	Fluoxetine	The degradation of fluoxetine is enhanced by the presence of ferrous ions that react with the sonogenerated H_2_O_2_.	[40]
Fe^2+^ (90 µmol L^−1^)	375 kHz	Ampicillin	The degradation and mineralization of ampicillin are enhanced by the presence of ferrous ions due to the Fenton reaction using the H_2_O_2_ coming from the sonochemical system.	[3]
Fe^2+^ (1.0, 3.0, and 5.0 mg L^−1^) and Fe^3+^ (1.0 mg L^−1^)	375 kHz	MO, ACE, and CIP	Acceleration of the MO and ACE degradation by adding Fe^2+^ by taking advantage of the sono-generated H_2_O_2_. However, an excess of Fe^2+^ leads to scavenging effects. Ferrous ions are more efficient than Ferric ions at accelerating the degradation of pollutants. Furthermore, CIP is complexed by Fe^3+^, limiting the performance of the sono-Fenton process.	This work

**Table 3:** Chromatographic conditions for analyses of CIP and ACE.

Pharmaceutical	Acetonitrile/Formic Acid (%/%)	Detection Wavelength (nm)	Flow (mL min^−1^)
Ciprofloxacin (CIP)	15/85	278	1.0
Acetaminophen (ACE)	15/85	243	0.6

## Data Availability

Data will be available by request through email to the corresponding authors.

## Supplementary Materials

The following supporting information can be downloaded at: https://www.mdpi.com/article/10.3390/molecules28031113/s1, Figure S1: Adsorption of MO on the natural mineral; Figure S2: (a) Adsorption of ACE on the natural mineral, (b) XRD pattern of the Colombian natural mineral, Figure S3: UV-Visible spectrum of CIP and its interaction with ferrous ions; Figure S4: UV-Visible spectrum of ACE and its mixture with ferric and ferrous ions; Figure S5: Mass spectrum of CIP; Figure S6: Mass spectrum of Product 1; Figure S7: Mass spectrum of Product 2; Figure S8: Mass spectrum of Product 3. Figure S9: pseudo-first-order rate constant (k) determination example, Table S1: Values of pseudo-first-order constants (k) for the degradation of the target pollutants.

## Sample Availability

Samples of the compounds are not available from the authors.
