# Supplementary material for: Possibilities and Limitations of the Sono-Fenton Process Using Mid-High-Frequency Ultrasound for the Degradation of Organic Pollutants
Source: Molecules. 2023 Jan 22;28(3):1113. doi: 10.3390/molecules28031113 (PMC9919913; doi:10.3390/molecules28031113)
Supplement: Supplementary file 1 [file molecules-28-01113-s001.zip › molecules-2101986-supplementary.pdf]

Supporting Information

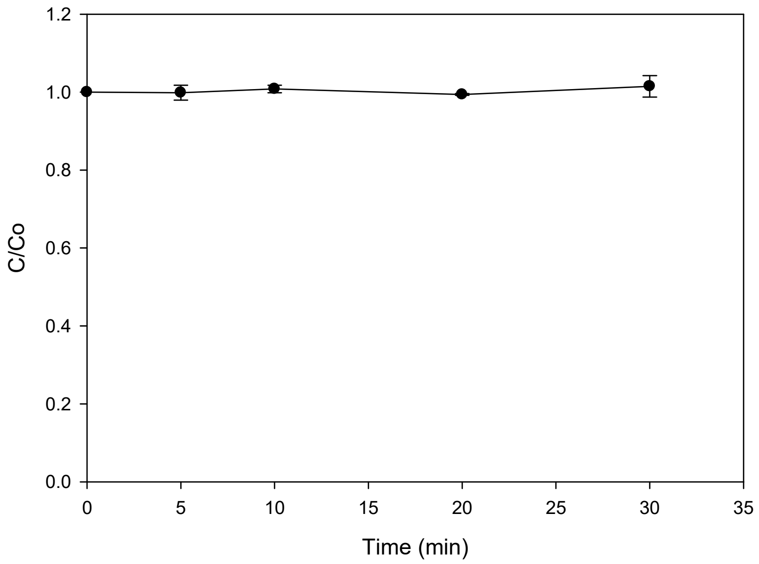

Figure S1. Adsorption of MO on the heterogeneous iron source (i.e., the natural mineral). Experimental conditions: $[MO]_{initial}$: 30.6 uM, [natural mineral]: 0.2 g L^{-1}.

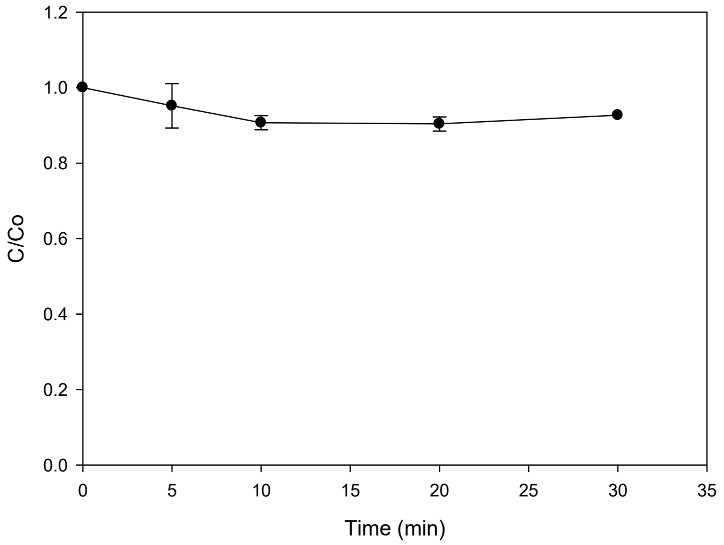

(a)

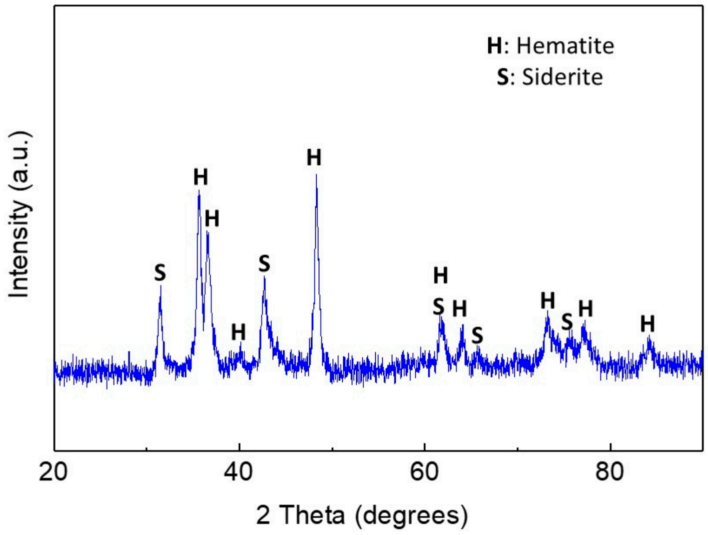

(b)

Figure S2. (a) Adsorption of ACE on the heterogeneous iron source (i.e., the natural mineral). Experimental conditions: $[ACE]_{initial}$: 30.6 uM, [natural mineral]: 0.2 g L^{-1}. **(b)** XRD pattern for the Colombian natural mineral.

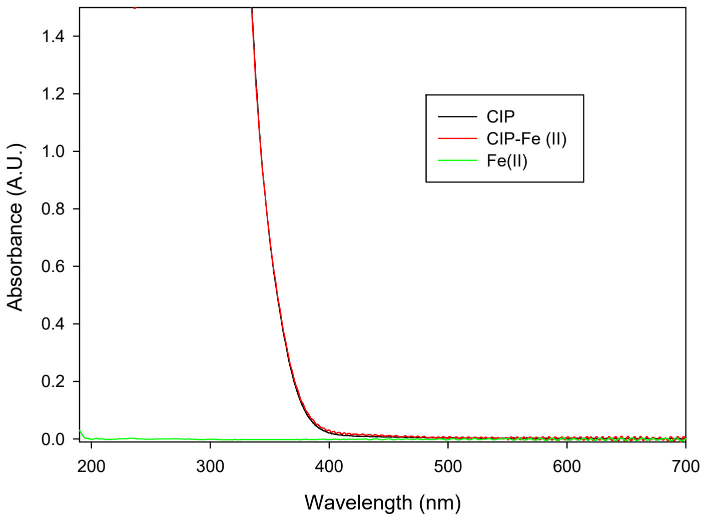

Figure S3. UV-Vis spectrum of CIP and its interaction with ferrous ions.

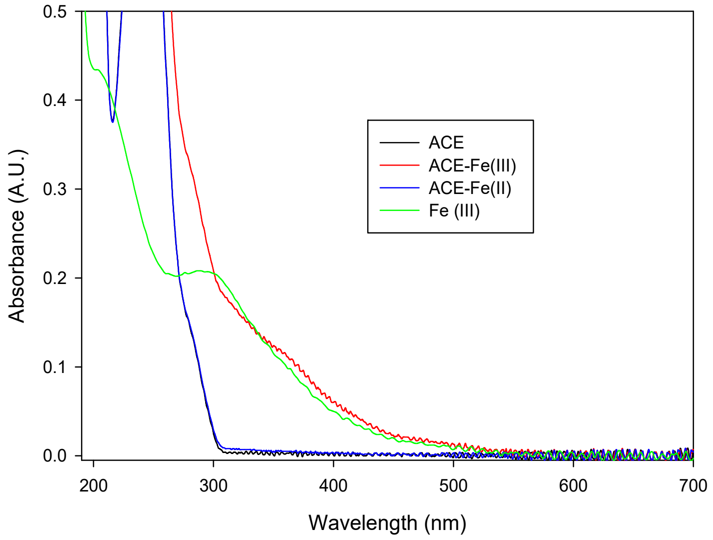

Figure S4. UV-Vis spectrum of ACE and its mixture with ferric and ferrous ions.

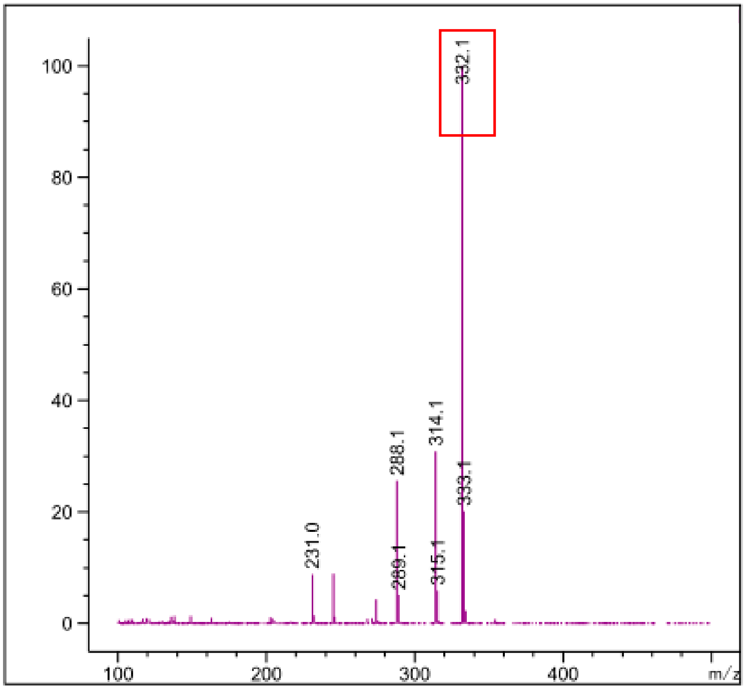

Figure S5. Mass spectrum of CIP.

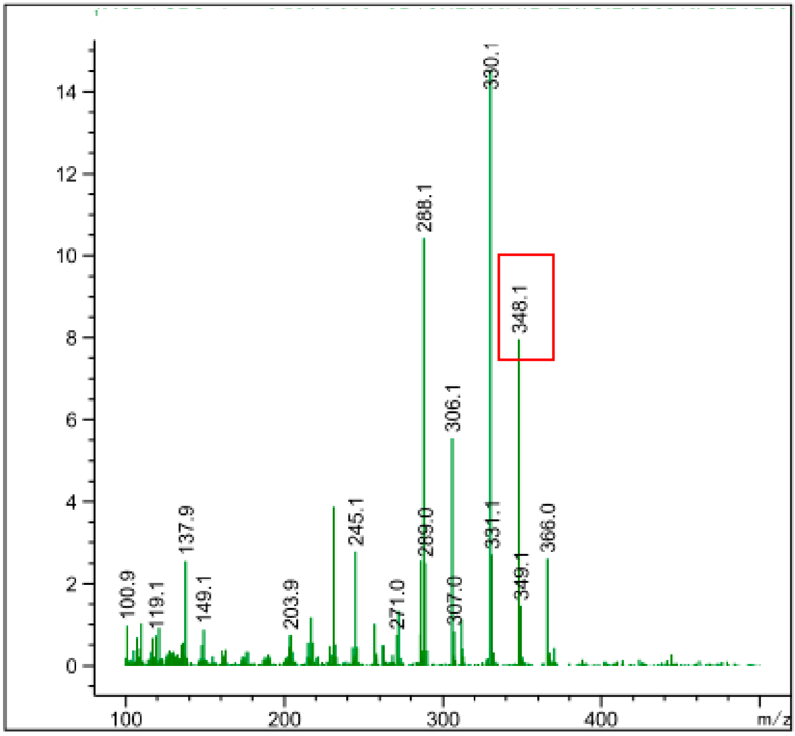

Figure S6. Mass spectrum of Product 1.

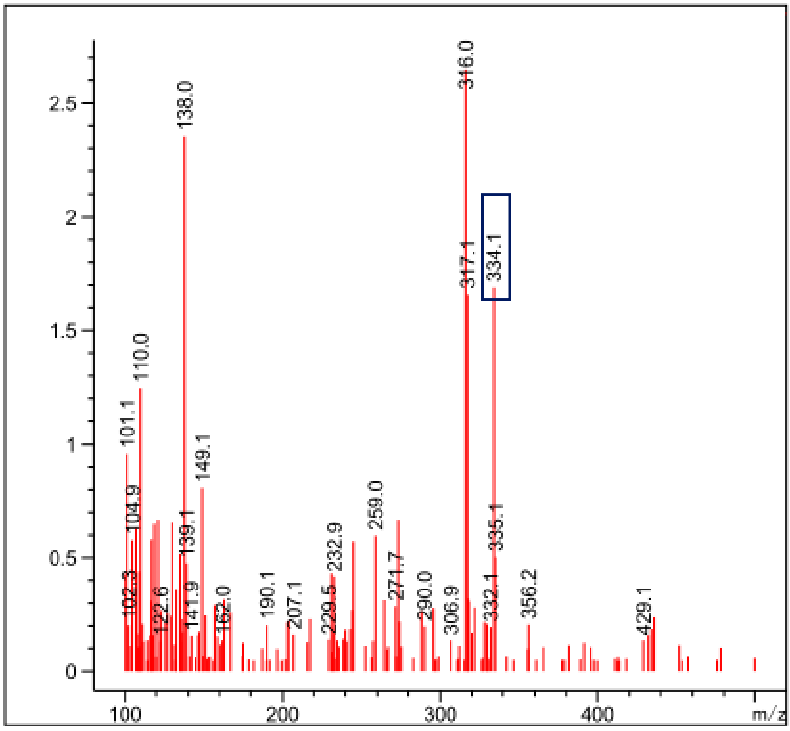

Figure S7. Mass spectrum of Product 2.

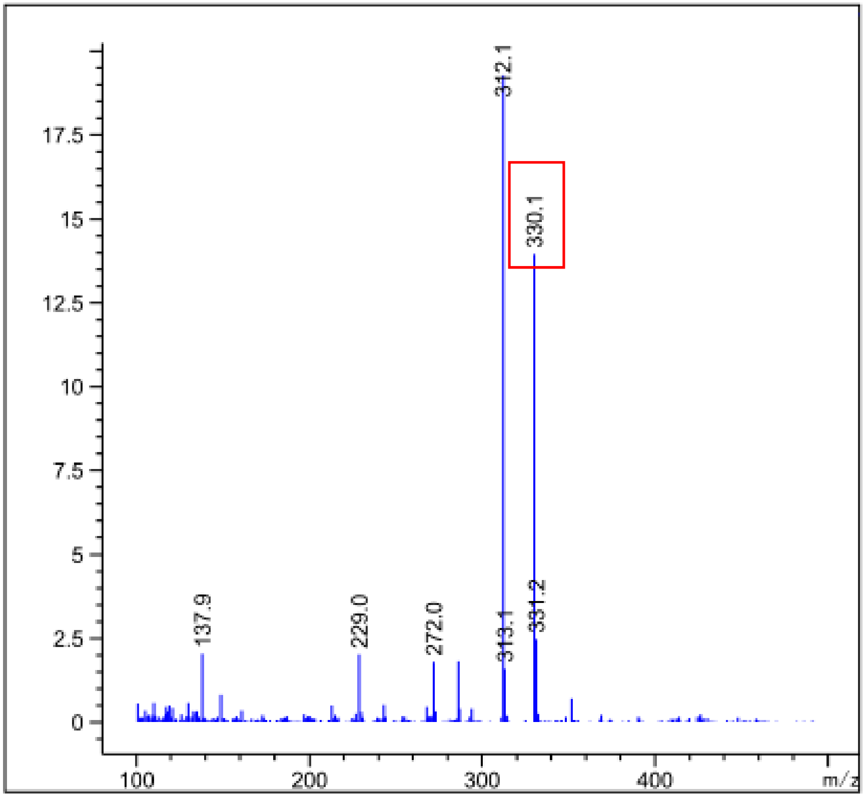

Figure S8. Mass spectrum of Product 3.

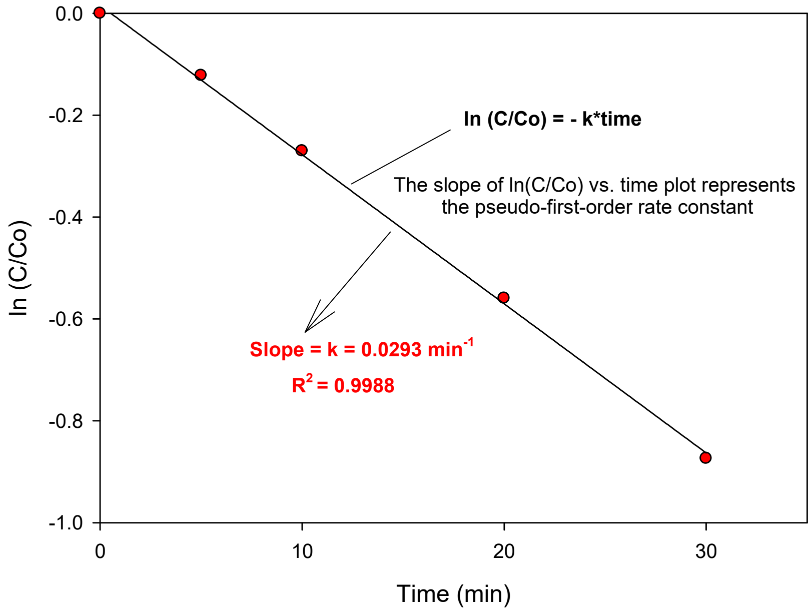

Figure S9. Example of the k-value determination from the ln C/Co vs. time plot. The data in this figure correspond to degradation of MO by ultrasound alone. *Experimental conditions:* f: 375 kHz, P: 34.4 W, $[MO]_{initial}$: 30.6 µmol L^{-1}, $pH_{initial}$: 5.6, and V: 250 mL.

Table S1. Values of pseudo-first-order constants (k) for the degradation of the target pollutants.

Experimental system	k-value (min^{-1})	Error for k (min^{-1})	R^2 (Correlation coefficient)
**MO (Figure 2a)**			
US	0.0293	0.0006	0.9988
US + mineral (0.02)	0.0280	0.0007	0.9981
US + mineral (0.20)	0.0306	0.0005	0.9992
**ACE (Figure 2b)**			
US	0.0282	0.0011	0.9959
US + mineral (0.20)	0.0257	0.0010	0.9954
**MO (Figure 3a)**			
0 ppm	0.0293	0.0006	0.9988
1 ppm	0.0382	0.0008	0.9987
3 ppm	0.0412	0.0010	0.9983
5 ppm	0.0366	0.0005	0.9994
**MO (Figure 3b)**			
US	0.0293	0.0006	0.9988
US + Fe (II)	0.0382	0.0008	0.9987
US + Fe (III)	0.0315	0.0004	0.9995
**ACE (Figure 4a)**			
US	0.0282	0.0011	0.9959
US + Fe (II)	0.0557	0.0023	0.9951
**CIP (Figure 4c)**			
US	0.0233	0.0012	0.9923
US + Fe (II)	0.0210	0.0066	0.7733
